# Effect of Acid Suppressants on Non–*Helicobacter pylori* Helicobacters Within Parietal Cells

**DOI:** 10.3389/fphar.2022.692437

**Published:** 2022-07-22

**Authors:** Masahiko Nakamura, Futa Murasato, Anders Øverby, Yosuke Kodama, Hirofumi Michimae, Kazuki Sasaki, Bram Flahou, Freddy Haesebrouck, Somay Y. Murayama, Shinichi Takahashi, Masayuki Uchida, Hidekazu Suzuki, Hidenori Matsui

**Affiliations:** ^1^ Ohmura Satoshi Memorial Institute, Kitasato University, Tokyo, Japan; ^2^ School of Pharmacy, Kitasato University, Tokyo, Japan; ^3^ Center of Education in Kongsvinger, Kongsvinger, Norway; ^4^ Department of Clinical Medicine (Biostatistics), School of Pharmacy, Kitasato University, Tokyo, Japan; ^5^ Department of Pathology, Bacteriology and Avian Diseases, Faculty of Veterinary Medicine, Ghent University, Merelbeke, Belgium; ^6^ Department of Fungal Infection, National Institute of Infectious Diseases, Tokyo, Japan; ^7^ Kyorin University School of Medicine, Mitaka, Japan; ^8^ Division of Research and Development, Meiji Dairies Corporation, Food Science Institute, Odawara, Japan; ^9^ Division of Gastroenterology and Hepatology, Department of Internal Medicine, Tokai University School of Medicine, Isehara, Japan

**Keywords:** non-*Helicobacter pylori* helicobacter, *Helicobacter suis*, urea breath test, vonoprazan, TUNEL, bilayer culture, coccoid form

## Abstract

We investigated the effect of increased pH induced by acid suppressants on the viability of non*–Helicobacter pylori* helicobacters (NHPHs) within parietal cell intracellular canaliculi and fundic glandular lumina by immunohistochemistry, electron microscopy, quantitative PCR, urea breath tests, and using a bilayer culture system. Three months before the experiment, mice were infected with the NHPH *H. suis* and then treated with famotidine (2 mg/kg body weight [BW], once daily), lansoprazole (30 mg/kg BW, once daily), or vonoprazan (20 mg/kg BW, once daily) for 3 days. Immunohistochemical studies using the TUNEL method, quantitative PCR analysis, and urea breath tests were performed. PCR analysis showed a decrease in the NHPH quantity after vonoprazan treatment. Urea breath tests revealed a significant decrease in the NHPH urease activity after vonoprazan, lansoprazole, and famotidine treatments for 3 days; however, 4 days after the treatment, urease activity reversed to the pretreatment level for each treatment group. Electron microscopy revealed an increase in the damaged NHPH after vonoprazan treatment. The TUNEL method revealed apoptotic NHPH within parietal cells after vonoprazan treatment. The bilayer culture results demonstrated that NHPH moved more quickly at a pH of 4.0 than at a pH of 3.0, 5.0, and 6.5, and electron microscopy revealed a change from the spiral form to the coccoid form under near-neutral pH conditions. We thus proposed that acid suppressants, especially vonoprazan, induce NHPH damage by altering pH.

## 1 Introduction

Although the prevalence of non-*Helicobacter pylori* helicobacters (NHPHs) is relatively low, compared with *Helicobacter pylori* (Hp), this bacterium has also been reported to be related to the formation of the MALT lymphoma, nodular gastritis, and chronic gastritis, as well as gastric cancer (Heilmann and Borchard, 1991; Nakamura et al., 2020; Yasuda et al., 2022). In addition, the number of reports of NHPHs has been increasing lately, partly because of the worldwide application of the Hp eradication regimen.

The infection of helicobacters into the gastric parietal cells was first detected by Bizzozero (1893) and Salmon (1896) in the mammalian stomach, but the pathological significance of the *Helicobacter* invasion into the potent acidic lumen is not well understood. These *Helicobacter* species are now thought to belong to NHPHs since Hp resides in the mucus layer and in the upper part of the fundic glands in human and primate stomachs, whereas NHPH can inhabit the lower layers of the fundic glands where many parietal cells are located, in addition to the mucus layer in several kinds of mammals including dogs, cats, pigs, and humans (Flahou et al., 2010; Nakamura et al., 2020). The morphology of parietal cells is well known to be greatly altered based on the extent of acid secretion levels, which can be altered through the administration of acid stimulants and suppressants (Soll and Walsh, 1979; Inokuchi et al., 1992; Scott et al., 1994; Schubert, 2015).

The membrane-recycling theory, i.e., the massive membrane flow from an endosomal compartment of tubulovesicle membranes to the apical secretory surface during strong acid secretion within the parietal cell, was postulated in the 1980s to explain how the morphology of parietal cells changes in response to changes in acid secretion levels (Forte and Zhu, 2010; Helander and Sundell, 1984; Calhoun and Goldenring, 1996). It is, thus, of clinical interest to understand the parietal cell morphology and NHPH viability during NHPH infection and acid suppression. We conducted this study to clarify the effects of the acid suppressants on NHPHs residing within parietal cells in mice with long-term NHPH infection. In this connection, we also investigated the influence of pH on the motility, survival, and morphology of NHPHs.

## 2 Materials and Methods

### 2.1 *In Vivo* Study

#### 2.1.1 Administration of Acid Suppressants

We obtained a urease-positive cynomolgus monkey stomach in 1993, and we perorally inoculated C3H mice with 0.1 ml of gastric mucosal and mucus homogenates. The bacteria were maintained *in vivo*, and this process was repeated every 12 months. Thereafter, 3 months before the present study, C57BL/6 mice were inoculated with gastric mucosal homogenates containing gastric mucosa and mucus from infected C57BL/6 mice (Nakamura et al., 2007).

We administered the following acid suppressants to these mice: famotidine (2 mg/kg body weight [BW], 1 × /day), lansoprazole (30 mg/kg BW, 1 × /day), and vonoprazan (20 mg/kg BW, 1 × /day), or physiological saline as a vehicle for 3 days through peroral intubation. Each group consisted of five mice. We determined the doses of agents following the references, mostly from animal experiments, because the dose for the animals cannot be calculated from the dose for humans, owing to the difference in the metabolism in animals. Famotidine dose was determined by Icatlo et. al (2000), which dealt with the *Helicobacter pylori* eradication in mice. The dose of lansoprazole was determined from the reports by Watanabe et al. (2004), where they used 3, 10, and 30 mg/kg BW once daily, and we selected 30 mg/kg BW. The dose of vonoprazan was determined from an animal study of the reports from the Pharmaceuticals and Medical Devices Agency of Japan (2004) and Sugano (2018).

#### 2.1.2 Intragastric pH Estimation

After the 3-day treatment, anesthesia with 2% isoflurane was administered *via* inhalation, cardiac blood sampling was performed, and the stomachs were removed and opened along the greater curvature. Intragastric pH was estimated by attaching a test strip (Advantec BTB, 6.2–7.7, 1–11, Toyo Roshi Co., Tokyo) to the surface of the fundic mucosa.

#### 2.1.3 Serum Gastrin Estimation

Blood samples from the mice were centrifuged at 11,000 X g for 5 min at 4°C. Serum gastrin I levels were estimated using an enzyme-linked immunosorbent assay kit specific to gastrin I (LS Bio, Seattle, WA, United States).

#### 2.1.4 Observation of Gastric Parietal Cells *via* Light Microscopy

After the stomach tissues were fixed with a neutral-buffered 10% solution, they were embedded in paraffin, and 4-μm sections were prepared using a microtome. The sections were stained with hematoxylin and eosin for histological examination.

#### 2.1.5 Immunohistochemical Examination and Electron Microscopy of *H. suis*

Immunohistochemical studies were performed using antibodies against the alpha subunits of H^+^/K^+^-ATPase (Smolka et al., 1982) and against the *H. suis* HsvA protein (Nakamura et al., 2020). Some tissues were fixed with Zamboni’s fixative for a better immunohistochemical reaction (Stefanini et al., 1967) and embedded in paraffin. After deparaffinization with xylene, a graded ethanol series, and phosphate-buffered saline (PBS), immunohistochemical studies using monoclonal antibodies were performed by confocal laser microscopy (Leica TCS NT, Leica Microsystems GmbH, Wetzlar, Germany).

We used electron microscopy to examine the biopsied specimens which were first fixed with 4% formaldehyde and 1% glutaraldehyde solution for 12 h, postfixed with 1% osmium tetroxide and ruthenium red solution, and then embedded in Epon 812 epoxy resin. Ultrathin sections were then cut, counterstained with a 3% aqueous solution of uranyl acetate for 30 min, and observed using a JEOL EX-II electron microscope (Akishima, Japan) at an accelerating voltage of 80 kV.

#### 2.1.6 TUNEL Method

Apoptosis was detected by terminal deoxynucleotidyl transferase (TdT)-mediated dUTP nick end labeling (TUNEL) using a TdT *in situ* detection kit that uses fluorescein for visualization (4812–30-K, R&D Systems; Minneapolis, MN, United States). After deparaffinization, sections were covered with a proteinase K solution, incubated for 15 min at 20°C, and washed three times with PBS for 5 min per wash. Then, the sections were immersed in a TdT labeling buffer for 5 min, allowed to react with a TdT labeling reaction mixture for 1 h at 37°C, washed once with phosphate-buffered saline (PBS) for 5 min, reacted with a TdT stop buffer, washed three times with PBS for 5 min per wash, and then left to react with a strep-fluorescein solution for 20 min at room temperature in the dark. Some sections were doubly stained with an anti-*H*.-*suis* antibody to clarify the relation to the TUNEL reaction and bacterial localization, embedded in PermaFluor (Thermo Scientific, Waltham, MA, United States), and observed by confocal laser microscopy.

#### 2.1.7 Estimation of *H. suis* by Quantitative Polymerase Chain Reaction

Real-time or quantitative polymerase chain reaction (qPCR) was used to amplify 16S rRNA and urease genes from the gastric samples. These reactions incorporated universal eubacterial primers (TM16S-27F and TM16S-1492R) for the 16S rRNA gene and the primers U430F and U1735R or U430F and U2235R for the urease genes. The cycling conditions for the 16S rRNA gene were as follows: initial denaturation at 94°C for 1 min, followed by 35 cycles of 94°C for 45 s (denaturation), 55°C for 30 s (annealing), and 72°C for 2 min (extension), followed by an additional extension step at 72°C for 5 min. The cycling conditions for the urease genes were initial denaturation at 94°C for 3 min, followed by 35 cycles of 94°C for 10 s, 52°C for 30 s, and 72°C for1.5 min; and a final extension step at 72°C for 5 min.

The conditions for qPCR of the urease gene were the same as those listed previously, but with the following modifications: annealing at 42°C for 30 s and additional extension at 72°C for 2 min. Both strands were sequenced using the BigDye^®^ Terminator v3.1 Cycle Sequencing Kit (Applied Biosystems, Carlsbad, CA, United States) on an ABI PRISM^®^ 3100-Avant Genetic Analyzer (Applied Biosystems). Sequences were analyzed using NCBC BLAST.

qPCR was performed using a Bio-Rad iCycler iQ Detection System (Bio-Rad Laboratories, Inc., Hercules, CA, United States) with SYBR Green fluorophores (Bio-Rad Laboraties, Inc.). Reactions were performed in a total volume of 20 μL, which included 10 µL of 2× SYBR Green PCR Master Mix (Bio-Rad Laboraties, Inc.), 5 µL of each primer at a concentration of 5 μM, and 1 µL of the template DNA. A 112-bp fragment of the 16S rRNA gene was amplified using the primer pair HeilF (5′-AAG TCG AAC GAT GAA GCC TA-3′) and HeilR (5′-ATT TGG TAT TAA TCA CCA TTT C-3′). Data analysis was performed using an iCycler iQ real-time detection system (Bio-Rad Laboraties, Inc.) (Nakamura et al., 2020).

#### 2.1.8 Estimation of the Urease Activity by Urea Breath Tests

After the administration of one of three acid suppressants or physiological saline as a vehicle for 3 days after the end of the treatments, 20 mice fasted for 8 h were perorally administered a solution of powder from a urea tablet and sterile water (4 mg/mouse) containing ^13^C-urea. The mice were then housed in a desiccator, from which their expired air was collected in a breath-sampling bag using a tube and an aspiration pump (Uchida et al., 2005). ^13^CO_2_ levels in the expired air were measured using an infrared spectrometer at appropriate intervals for 20 min. Thereafter, 4 days after the administration of acid suppressants, the same mice were used again for the estimation of the urease activity.

### 2.2 Culture Study

*H. suis*, *H. heilmannii*, and *H. felis* were kind gifts from Drs. B. Flahou and F. Haesebrouck. We cultured the bacteria *via* the bilayer culture method outlined by Flahou et al. (2010) and then estimated the motility, urease activity, and morphological changes of the bacteria under different pH conditions of the culture medium by vital microscopy. We evaluated the urease activity of cultured NHPHs using 100 μL of the rapid urease test (RUT) solution per well of a microplate to which was added 1uL of cells collected after plate growth. Color change was determined after 15 min using a plate reader (ChroMate-6 Microplate reader, Microtec Co., LTD).

To estimate motility, we examined specimens *via* light microscopy. Glass slides were prepared with a loopful of growth from a nutrient agar subculture mixed with a drop of sterile distilled water on one end and another loopful of growth from the same subculture in a drop of peptone water on the other end. Both ends of the slides were covered with a cover glass and were examined under a light microscope with a ×40 objective.

Some specimens from each treatment group were observed by immunohistochemistry using an antibody against *Helicobacter* (Nakamura et al., 2007) and electron microscopy using the same method described earlier.

### 2.3 Statistics

All data are expressed as the mean ± standard deviation. Two-factor ANOVA was mostly used in this study. One-factor ANOVA and Bonferroni correction as a *post hoc* test were also used in some parts. The significance level was determined as less than 5%.

## 3 Results

### 3.1 *In Vivo* Study

#### 3.1.1 Effect of Acid Suppressants on the Gastric pH

**FIGURE 1:**
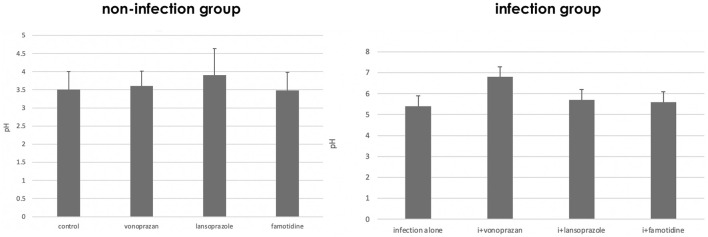
Alteration of gastric pH by acid suppressants and NHPH infection. A significant interaction was found between infection and acid suppressants in the gastric mucosal pH (F = 3.04 and *p* = 0.043 by two-way ANOVA). In the infection group, vonoprazan treatment induced the highest increase in intragastric pH. i means infection in the left graph.

In the NHPH infection group, administration of vonoprazan induced the highest increase in the intragastric pH both in the infected groups 3 days after the administration (Figure 1). A significant interaction was found between infection and acid suppressants in the gastric mucosal pH.

#### 3.1.2 Effect of Acid Suppressants on the Serum Gastrin Concentration

**FIGURE 2:**
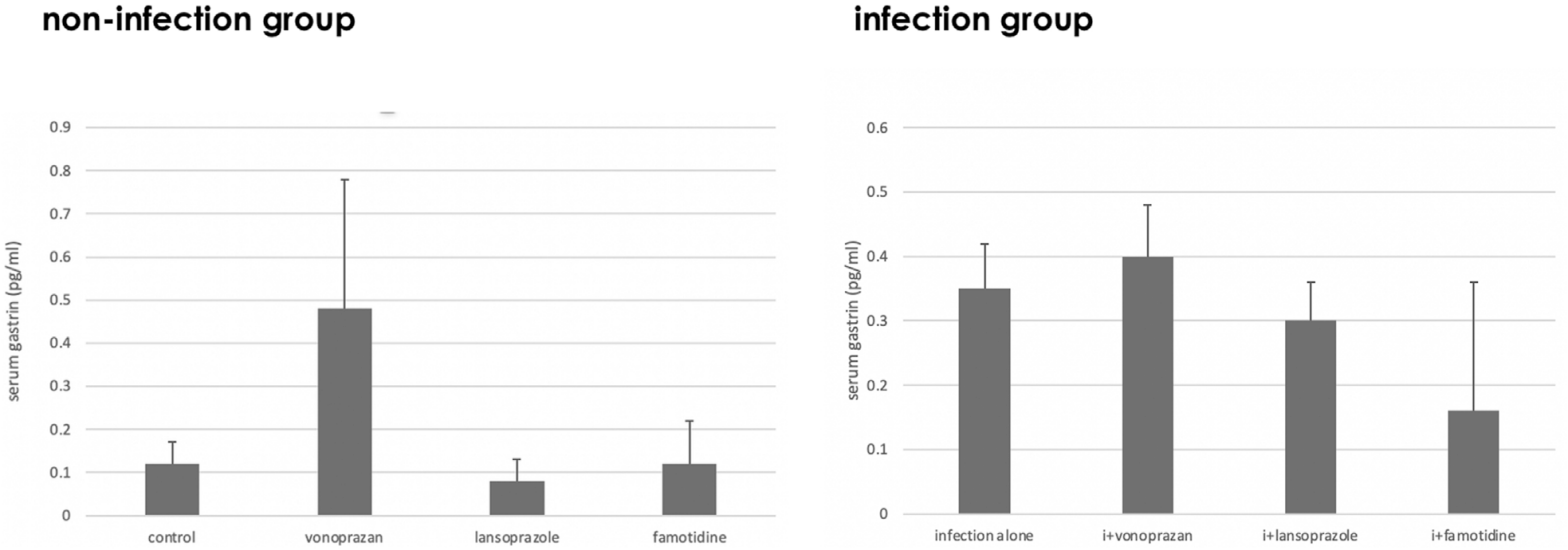
Alteration of the serum gastrin level by acid suppressants and NHPH infection. A significant interaction was found between infection and acid suppressants in the serum gastrin level (F = 5.61; *p* = 0.003).

As to the serum gastrin level, a significant interaction was found between infection and acid suppressants (Figure 2). The highest serum gastrin level was detected in the vonoprazan-treated mice both in non-infected and infected groups.

#### 3.1.3 Effect of Acid Suppressants on Parietal Cell Morphology

**FIGURE 3:**
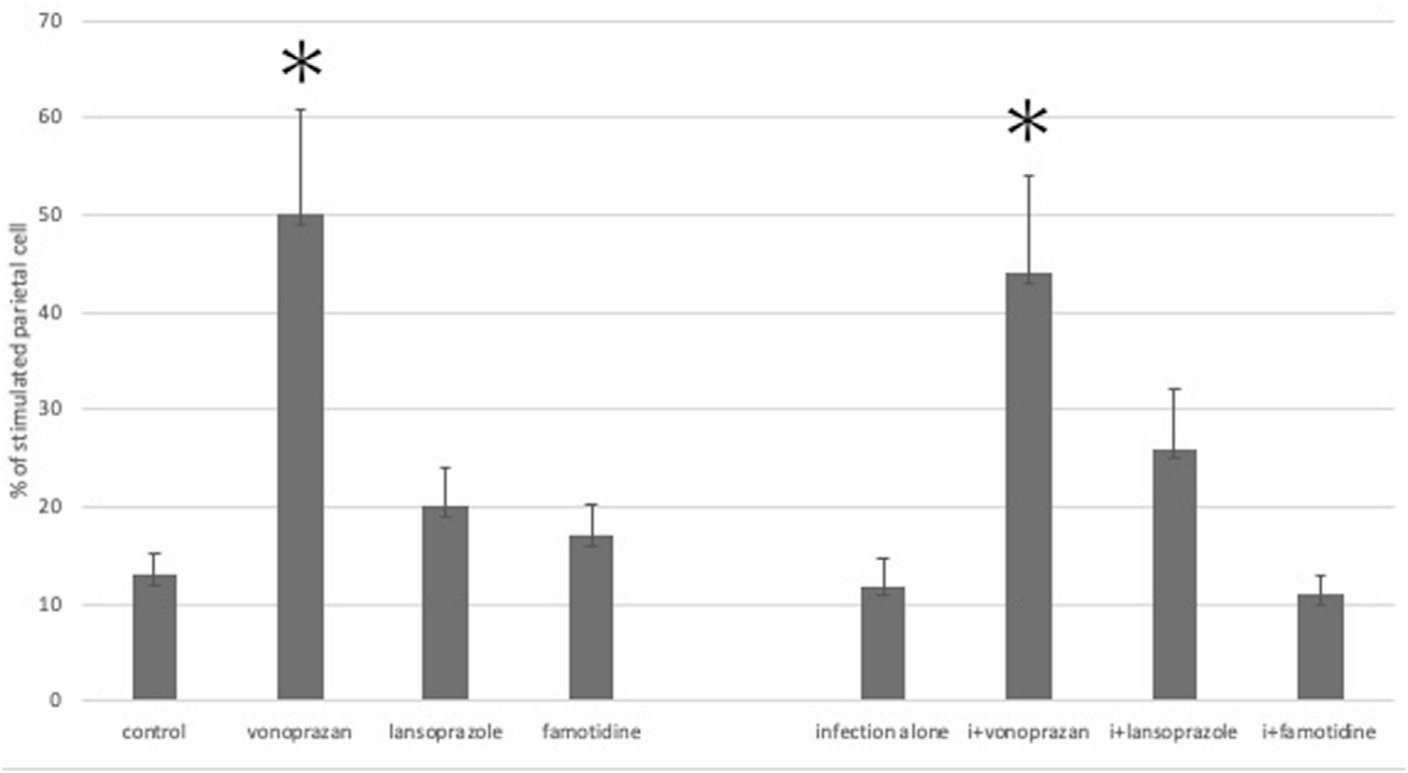
Alteration of the parietal cell structure by acid suppressants and NHPH infection. No significant interaction was found between infection and acid suppressants in the percentage of the stimulated parietal cell (F = 1.54; *p* = 0.224). By the *post hoc* analysis, the vonoprazan-treated group showed a significant increase in the stimulated parietal cell in both non-infection and infection groups (*p* < 0.05).

**FIGURE 4:**
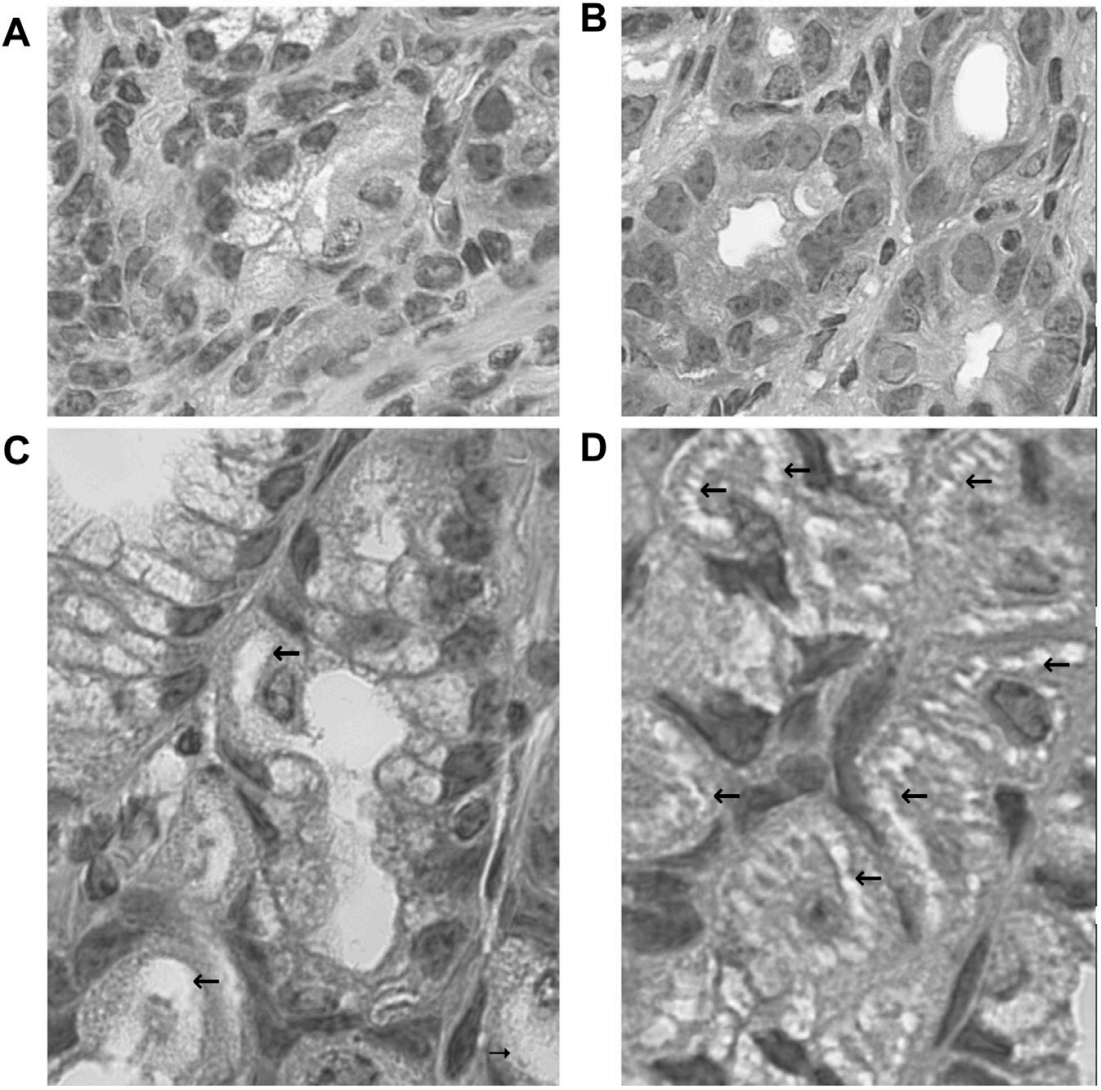
Morphological changes of fundic glandular cells by acid suppressants under NHPH infection. HE stains **(A)** the control group fundic mucosa, X 400. **(B)** In famotidine-treated mice, no significant changes in the parietal cell were detected in the body portion of the fundic gland, X 400. **(C)** In the lansoprazole-treated mice, many parietal cells having very enlarged intracellular canaliculi (arrows) were seen, X 600. **(D)** In the vonoprazan-treated mice, most parietal cells had the enlarged intracellular canaliculi (arrows) surrounding the nucleus, X 600.

Compared to the control group, vonoprazan-treated mice showed a significant increase in stimulated parietal cells, which are defined as having an enlarged intracellular canaliculus, whereas no significant differences were observed in the other treatment groups (Figures 3, 4).

#### 3.1.4 Effect of Acid Suppressants on the Localization and Shape of Non–*Helicobacter pylori* Helicobacters

**FIGURE 5:**
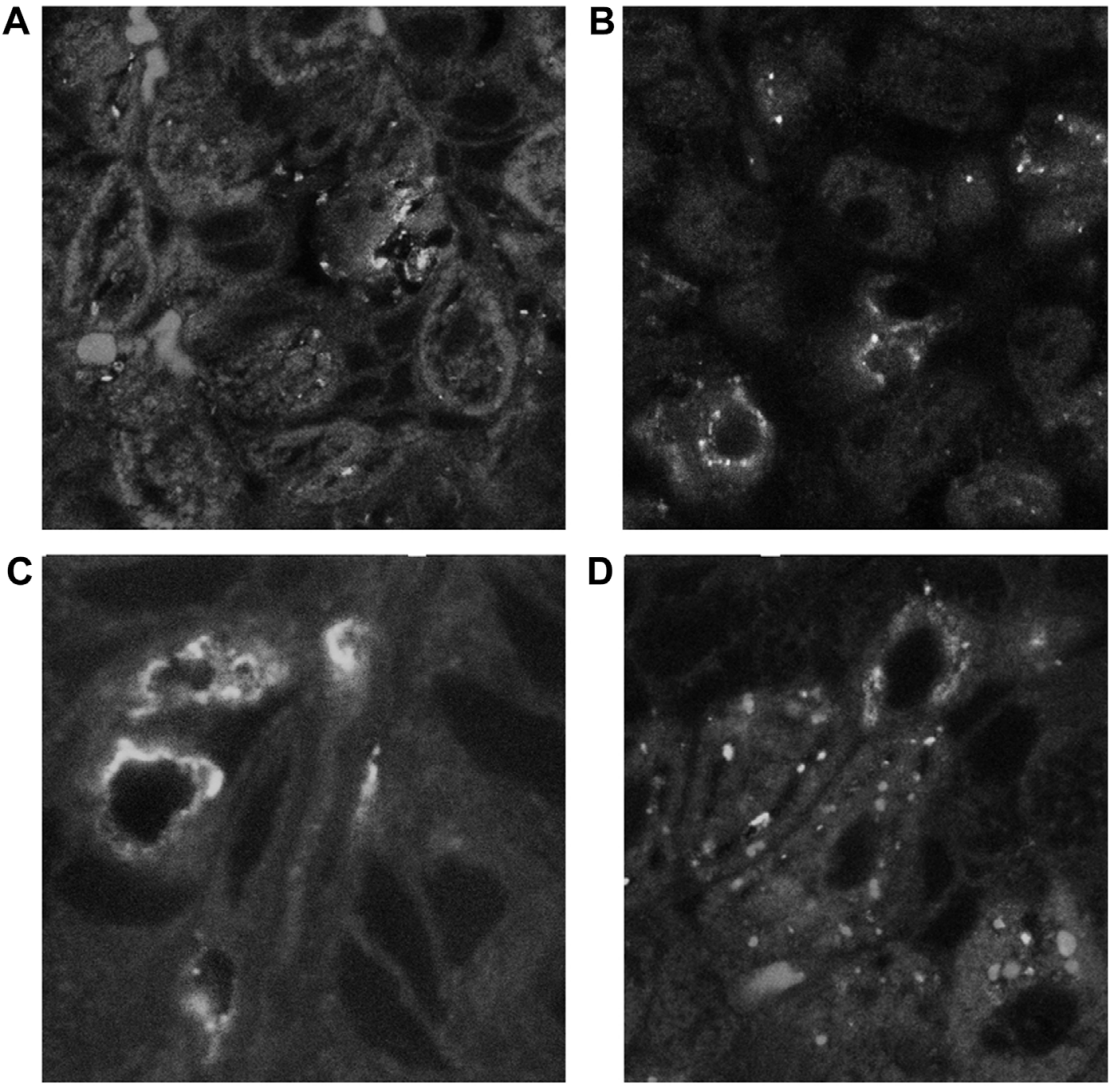
Alteration of NHPH localization in the fundic gland in the infection group by acid suppressants by indirect fluorescent immunohistochemistry using an anti-HsvA antibody. **(A)** In the infection-alone group, linear-shaped NHPHs were mostly found within the intracellular canaliculi of the parietal cells and in the gastric glandular lumen, X 400. **(B)** In the famotidine-treated mice, many spiral-shaped NHPHs were seen in the intracellular canaliculi of the parietal cells and in the gastric glandular lumen, X 400. **(C)** In the lansoprazole-treated mice, many spiral-shaped NHPHs were seen in the enlarged intracellular canaliculus of the parietal cells and in the gastric glandular lumen, X 600. **(D)** In the vonoprazan-treated mice, the round-shaped NHPHs were mostly seen in the intracellular canaliculus of the parietal cells, X 400.

Spiral-form NHPHs were observed in the intracellular canaliculi of the parietal cells of famotidine-treated, lansoprazole-treated, and control groups by fluorescent immunohistochemistry. In vonoprazan-treated mice, the shape of NHPHs was mostly altered from a spiral form to a round form (Figure 5).

#### 3.1.5 Effect of Acid Suppressants on the Number of Non–*Helicobacter pylori* Helicobacters Shown by Polymerase Chain Reaction

**FIGURE 6:**
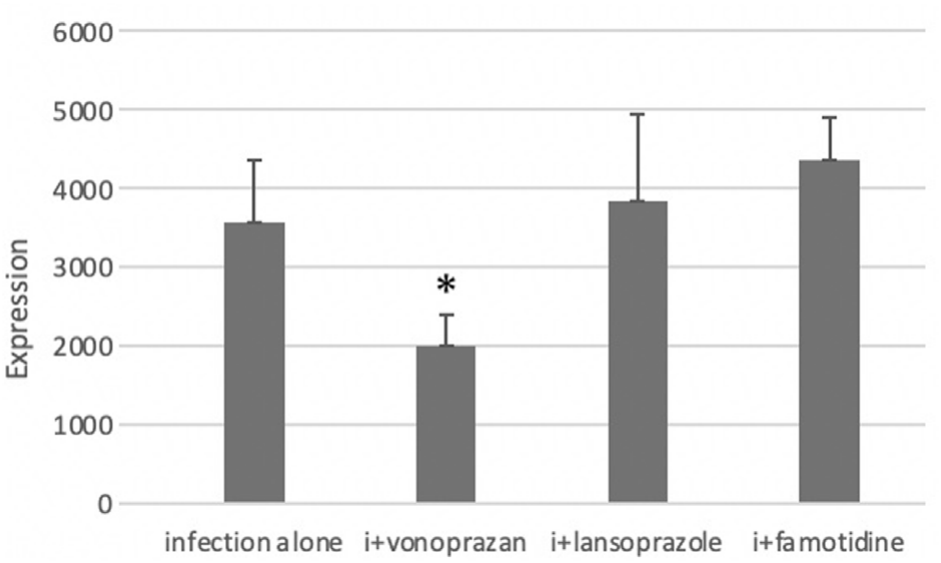
Effect of acid suppressants on the NHPH number by PCR. PCR analysis showed significant difference between infection alone and administered agents (F = 5.33 and *p* = 0.008 by one-way ANOVA). *Post hoc* analyses (Bonferroni correction) revealed that the infection-alone group is significantly larger than the infection + vonoprazan group (*p = 0.008). Decrease in NHPH in the vonoprazan-treated mice, but significant interaction was found between infection and administered agents in NHPHs (F = 7.17; *p* = 0.008).

PCR analysis showed a significant decrease in NHPHs in the infection and vonoprazan-treated mice, compared with the infection-alone group (Figure 6).

#### 3.1.6 Vonoprazan Induced TUNEL-Detectable Apoptosis

**FIGURE 7:**
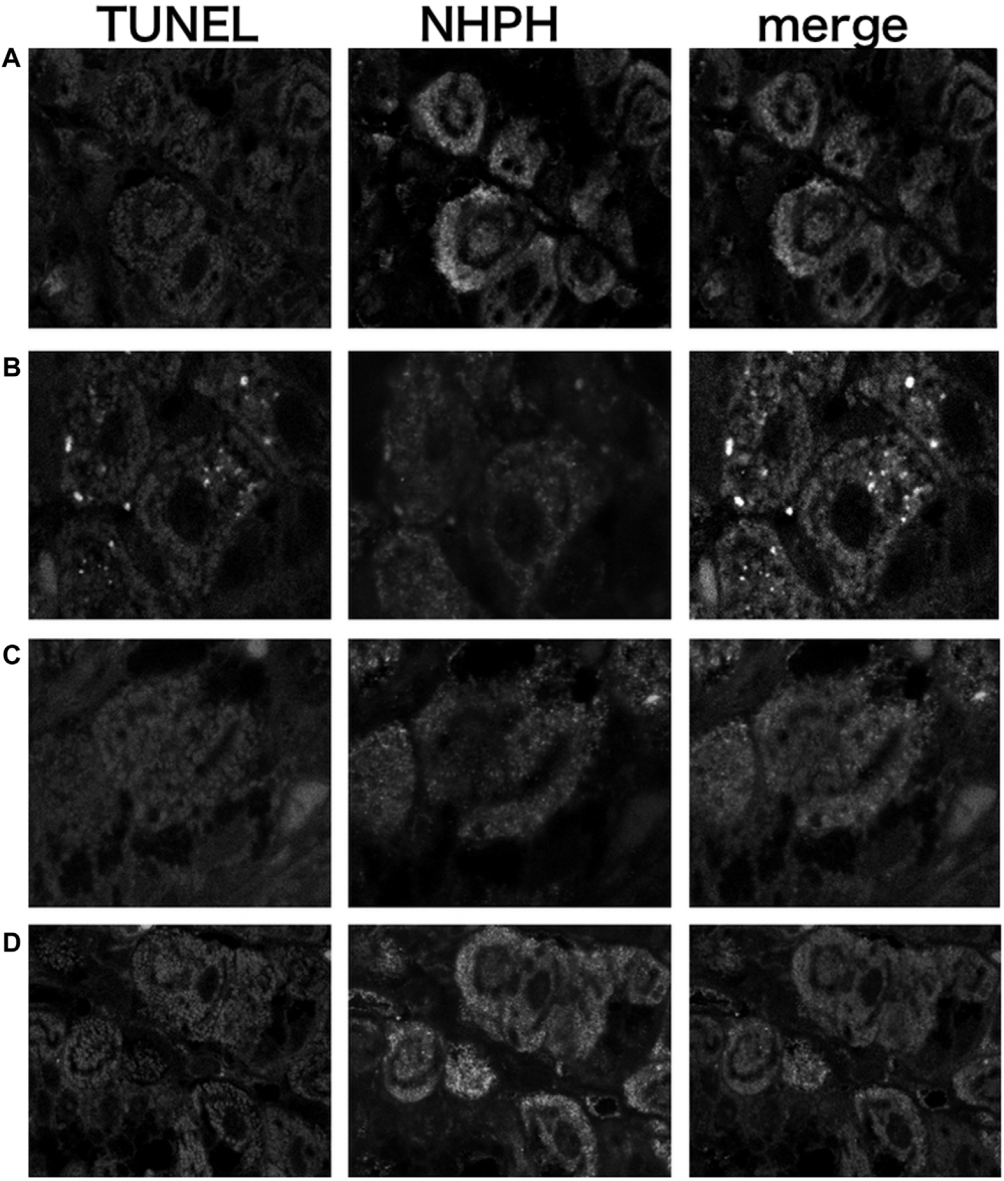
Localization of apoptotic NHPHs in the infection group by acid suppressants by the TUNEL method X 200. **(A)** In the infection-alone group, no apoptosis was detected. **(B)** In the vonoprazan-treated group, apoptotic NHPHs were detected within the intracellular canaliculi of the parietal cells. **(C)** In the lansoprazole-treated group, many NHPHs were found, but no apoptotic reaction was recognized. **(D)** In the famotidine-treated group, NHPHs in the parietal cells were negative for the TUNEL reaction.

**FIGURE 8:**
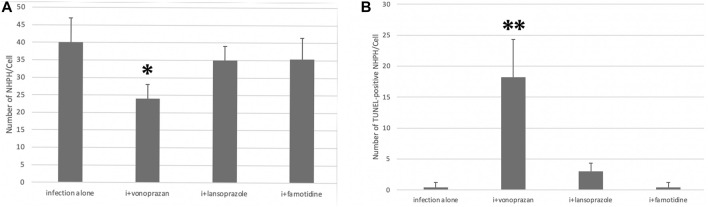
Quantitative analysis of all NHPHs and apoptotic NHPHs by using acid suppressants by immunohistochemistry. **(A)** Significant difference was found between infection alone and administered agents in the NHPH number (F = 6.14; *p* = 0.005 by one-way ANOVA). *Post hoc* analyses (Bonferroni correction) revealed that the infection-alone group is significantly larger than the infection + vonoprazan group (*p = 0.004). **(B)** Significant difference was found between infection alone and administered agents in the TUNEL-positive NHPH number (F = 28.1; *p* < 0.001 by one-way ANOVA). *Post hoc* analyses (Bonferroni correction) revealed that the infection + vonoprazan group is significantly larger than the infection-alone group (**p < 0.001).

The TUNEL method revealed that only the vonoprazan treatment induced the apoptosis of NHPHs within the parietal cells (Figure 7). The results of the quantitative analysis showed a significant decrease in NHPHs within the parietal cell in the vonoprazan-treated group, compared with the infection-alone group. In addition, the TUNEL-positive apoptotic NHPHs within the parietal cells significantly increased in the vonoprazan-treated group (Figure 8).

#### 3.1.7 Electron Microscopic Observation of Non–*Helicobacter pylori* Helicobacters Damaged by Vonoprazans

**FIGURE 9:**
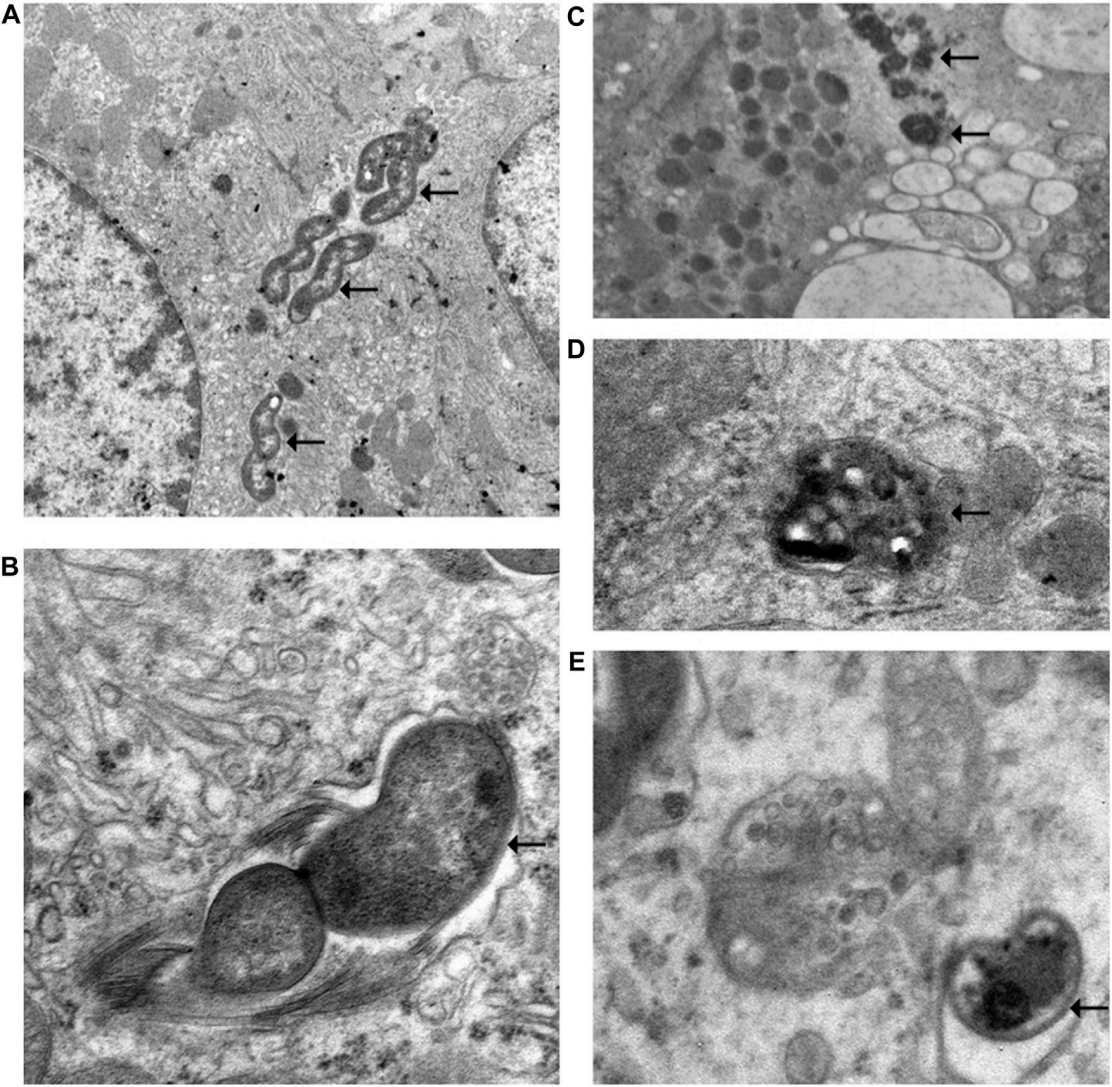
Electron microscopic observation of NHPH in infection-alone and infection + vonoprazan-treated mouse gastric parietal cells. **(A)** In infection alone mouse, many NHPHs were seen within the intracellular canaliculus of the parietal cell in the gastric mucosa, X 8,000. **(B)** In a higher magnification, flagella were recognized adjacent to the NHPH cell body in the intracellular canaliculus, X 24,000. **(C)** In the infection + vonoprazan-treated mouse, the bacterial cell body became condensed and irregular (arrows) in the intracellular canaliculus of the parietal cell, X 8,000. **(D,E)** Many bacilli were found in the secondary lysosome in the infection + vonoprazan-treated group, X 8,000.

The electron microscopic cytochemical observations showed that the vonoprazan treatment of NHPH-infected mice induced condensation and deformity of the NHPHs within the intracellular canaliculi of the parietal cell (Figure 9). Many secondary lysosomes having NHPHs inside were found in the cytoplasm of the parietal cells in the infection and vonoprazan-treated mice.

#### 3.1.8 Effects of the Acid Suppressants on the Urease Activity by the Urea Breath Test

**FIGURE 10:**
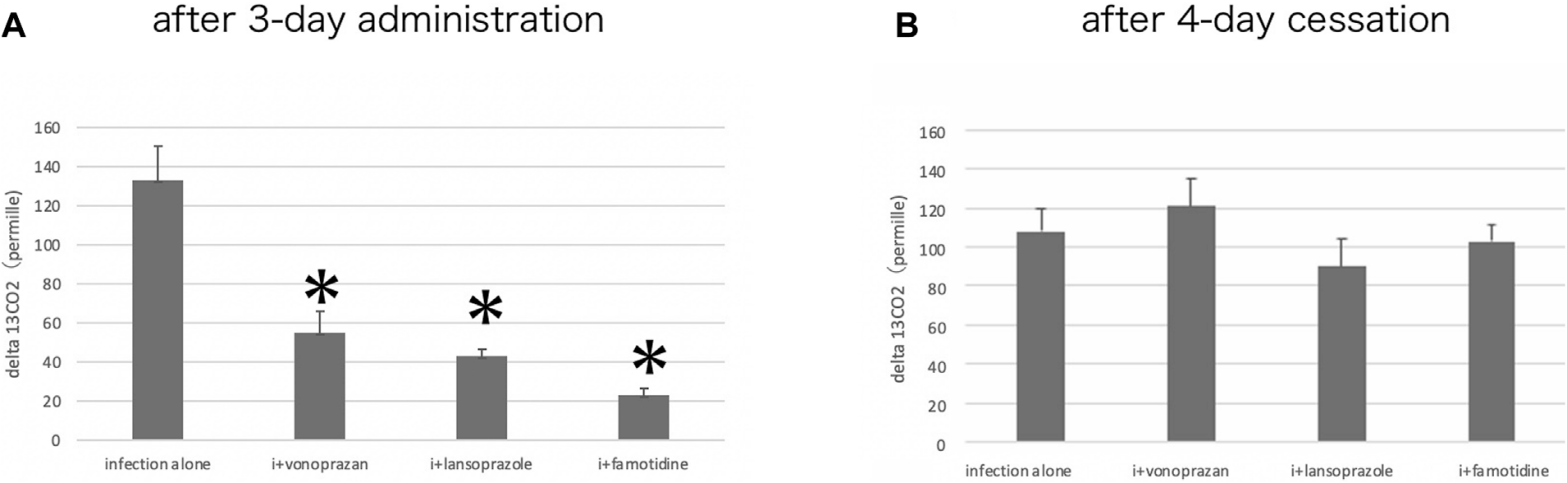
Alteration of the urease activity by acid suppressants. **(A)** By the urea breath test, a significant interaction was found between infection alone and administered agents (F = 47.1; *p* = 0.001 by one-way ANOVA). *Post hoc* analyses (Bonferroni correction) revealed that the infection-alone group is significantly larger than the other three groups (*p < 0.001) after three-day administration. **(B)** Cessation of the administration for 4 days resulted in the recovery of the urease activity in all drug-administered groups.

The urea breath test revealed that vonoprazan, lansoprazole, and famotidine each significantly decreased the Cmax of ^13^CO_2_ excretion compared to the untreated infection-alone group, signaling a decrease in the urease activity. Then, 4 days after the completion of the acid suppressant treatments, the urease activity showed no differences among infection-alone and all of the drug-treated groups (Figure 10). This means that the significant suppression of the urease activity took place by 3 days of administration of acid suppressants, and the urease activity of the bacteria could recover if no drugs were administered.

### 3.2 Culture Study

#### 3.2.1 Effect of the Culture Medium pH on the Morphology of Non–*Helicobacter pylori* Helicobacters

**FIGURE 11:**
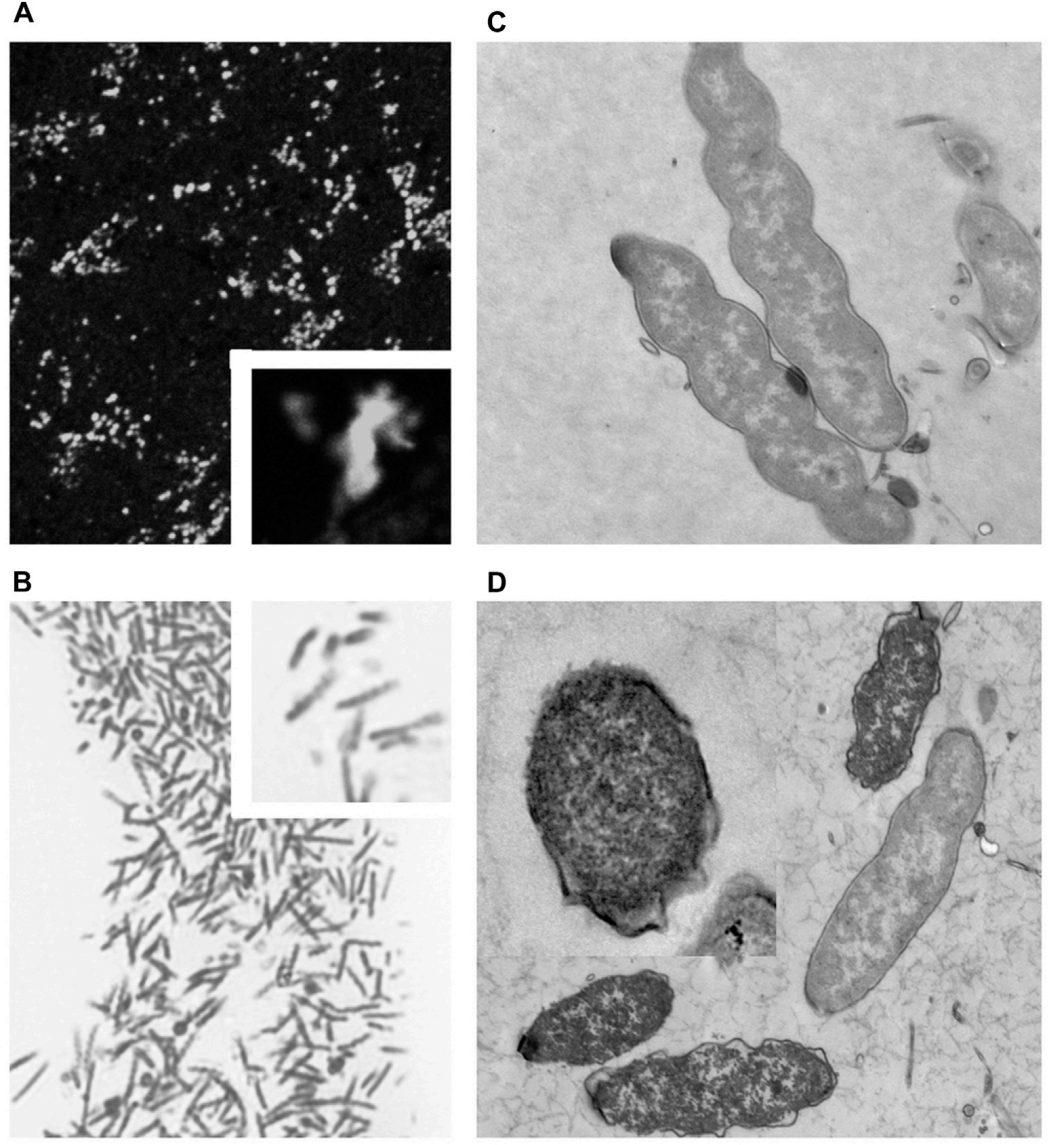
Fluorescent, light, and electron microscopic observations of the cultured NHPH **(A)** By fluorescent immunohistochemical observation of the cultured NHPH using the HsvA antibody, many bacteria were observed in the incubation medium, X 400. Inset X 1800. **(B)** By light microscopic observation of the toluidine-blue stained Epon-embedded 2-µM section, many spiral bacilli were observed, X 600. Inset X 1800. **(C)** Electron microscopic observation of the cultured intact NHPH in pH 3. Intact bacilli were observed with flagella, X 15, 000. Ruthenium red *en bloc* staining. **(D)** Electron microscopic observation of the cultured NHPH in pH 6.5. Ruthenium red *en bloc* staining. The cytoplasm of some of the bacilli became condensed and homogeneous, and its plasma membrane became irregular, coinciding with the observation reported in the coccoid form, X 12,000, Inset x15, 000.

Many NHPHs showed the spiral form when the culture pH was 3.0 (Figure 11). Ruthenium red *en bloc* staining during electron microscopy revealed that the coccoid form bacilli were observed in addition to the intact form at a pH of 5.0.

#### 3.2.2 Effect of the Culture Medium pH on the Motility and Urease Activity of Non–*Helicobacter pylori* Helicobacters

**FIGURE 12:**
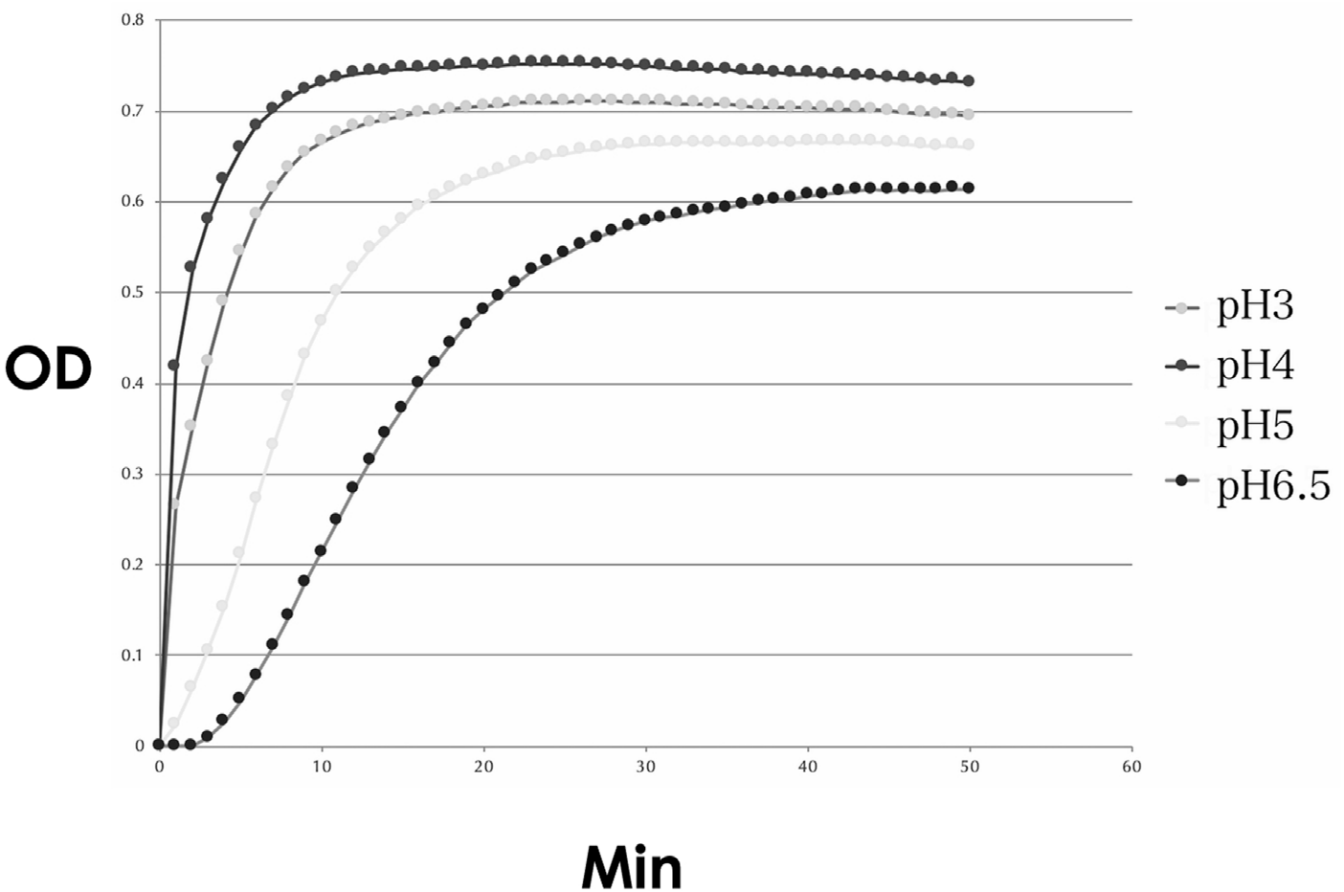
Urease activity of the cultured NHPH. Urease activity was found to be strongest at pH 4, followed by pH 3, and became weak at pH 5 and 6.5. OD: optical density.

The urease activity was the highest at a pH of 4.0, followed by that at a pH of 3.0 (Figure 12).

**FIGURE 13:**
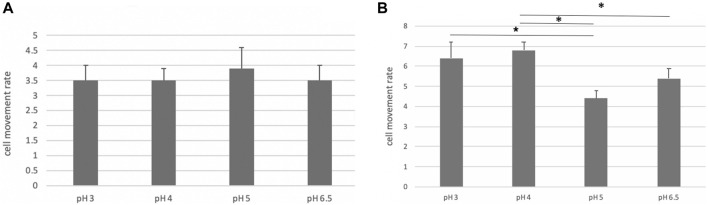
Motility of the cultured NHPH. On day 2, no significant difference was found, as shown in **(A)**, while a significant decrease in motility was detected at pH 5 and 6.5 on day 3 **(B)** by *post hoc* analysis (*<0.05).

The motility of the bacteria was decreased at pH values > 5.0, compared to that at pH of 3.0 and 4.0 (Figure 13). These data coincided with the above-mentioned *in vivo* study that vonoprazan administration with a pH of more than 6 induced the damage of NHPHs.

## 4 Discussion

As one of the most potent acid secreting cells in the human body, the parietal cells have fascinated many scientists for decades (Harms, 1910). The relationship between parietal cells and the formation of gastric ulcers has been investigated (Davenport, 1968). Schreiber et al. (2000) and Schreiber et al. (2007) used ultrafine double-barreled tip-sealed microelectrodes in their examinations of guinea pig stomachs and observed that the average glandular lumen pH was approximately 3.0 near the apical membrane of the intracellular canaliculus of the parietal cells and 4.6 at the crypt outlet, although Chu et al. (1999) reported that alkaline secretion protected the gastric mucosal surface by *in vivo* confocal imaging.

Regarding the alteration of parietal cells’ morphology by the administration of acid suppressants, Stolte et al. (1992) demonstrated the pseudohypertrophy of parietal cells treated with omeprazole. They speculated that this phenomenon occurs due to hypergastrinemia since this change in morphology was not observed in post-antrectomy patients who had few gastrin-secreting cells. Karasawa et al. (1988) reported the vacuolation of parietal cells by omeprazole, and they postulated that the interaction of this structure was due to the direct acid-inhibiting effect of omeprazole. Inokuchi et al. (1992) reported parietal cell damage by omeprazole, and Scott et al. (1994) described the structural change of parietal cells to an enlarged intracanalicular space, following the administration of omeprazole, which is similar to the stimulated state of parietal cells. The secretory state is the opposite.

This study used lansoprazole (a proton pump inhibitor), vonoprazan, potassium competitive acid blockers (a new type of acid antisecretory agent), and famotidine, an H_2_-receptor antagonist. Treatment with vonoprazan and lansoprazole induced a similar enlargement of intracellular canaliculi in parietal cells. Famotidine did not cause a significant change in the morphology of parietal cells, which is consistent with a report by Scott et al. (1994).

In our study, the serum gastrin level did not significantly alter 3 days after the drug administration. Larson et al. (1986) reported the induction of hypergastrinemia by omeprazole administration daily for 20 days in dogs, which suggests our study was performed in a too short a period to induce the changes in the serum gastrin level. This may also mean that the morphological changes of parietal cells occurred by other mechanisms than hypergastrinemia because these changes were observed without hypergastrinemia in this study.

During our histological observations, we were interested in the interaction of this enlarged intracellular canalicular lumen and the NHPHs preferentially inhabiting this lumen. As mentioned in the Introduction, the invasion of helicobacters into gastric parietal cells was first detected by Bizzozero (1893) and Salomon (1896); however, the pathological significance of the invasion into this potent acidic lumen is not well understood. In 2005, De Bock et al. reported the loss of gastric parietal cells and the abundance of bacteria by *H. felis* infection in Mongolian gerbils, demonstrating the deteriorating effect on acid secretion by NHPHs (De Bock et al., 2006). Liu et al. (2014) described the loss of parietal cells and the induction of gastric metaplasia in BALB/c mice infected with *Helicobacter heilmannii*. In humans, *Helicobacter heilmannii* infection has been reported to be rather mild and not strongly related to gastric atrophy and intestinal metaplasia (Heilmann and Borchard, 1991; Stolte, et al., 1997; Nakamura, et al., 2020).

As to the severity of gastric mucosal atrophy, we did not estimate the parietal cell density in this study; mild gastric atrophy with intestinal metaplasia was detected in our other study using *H. suis* infection (Rimbara et al., 2021). This could explain more effectiveness of acid suppressants in the infection group. The cytokines including IL-1beta could also play some role in the infection group to increase acid suppression (El-Omar, 2001).

Acid inhibition by lansoprazole is known to be slower than that of famotidine and vonoprazan, but 1 day after the treatment, intragastric pH is reported to increase from 2.11 to 3.57, and three-day treatment is thought to be enough to compare with other agents (Bell and Hunt, 1996).

In the present study, strong acid suppression was shown to be accompanied by a decrease in the NHPH urease activity since significant differences were produced by treatments with vonoprazan, lansoprazole, or famotidine. This could be explained as an adaptation to the increase of the environmental pH where a strong urease activity is not necessary for the survival of the bacteria. However, the number of NHPH cells was slightly decreased in the vonoprazan-treated group, and about half of the NHPH cells in this group became apoptotic. The main difference between vonoprazan and the other two agents is the potency of acid suppression, which is thought to be closely related to the rate of apoptosis if vonoprazan does not have a direct effect. This hypothesis is supported by the results of our culture study: NHPHs adapted best to a pH of 3.0 or 4.0, and vonoprazan administration altered the gastric glandular surface pH by more than 6. To exclude the direct interaction of acid suppressants to NHPH, the PCR analysis of cultured NHPHs in different pH incubation media is thought to be quite helpful and should be tried in a future study. **I**n addition, glycans and glycan-associated receptors were recently reported to have interacted with NHPH in the gastric mucosa. These factors could play some role in pH changes (Matos et al., 2021).

Lansoprazole was reported to have a direct suppressive effect on *H. pylori* other than acid suppression acting on the parietal cell (Iwahi et al., 1991) and could have some influence on NHPHs.

The relationship between apoptosis and coccoid formation has not been clarified in the case of *Helicobacter pylori,* although the alteration from the spiral form to the coccoid form has been extensively investigated (Ogata et al., 1998, Azevedo et al., 2007). The coccoid formation was initially considered to be a viable but non-culturable form capable of surviving under unfavorable conditions. However, studies of *H. pylori* have suggested that coccoid formation represents a non-viable, degenerative form (Vijayakumari et al., 1995). In the present study, NHPHs affected by vonoprazan showed a structure similar to that of the coccoid form of *H. pylori*. Four-day cessations of the acid suppressant administration were shown to recover the urease activity, suggesting some of the damage of NHPHs by low pH could be reversible. PCR analysis may clarify this point, although the damaged NHPH other than viable NHPH could be also counted.

*Escherichia coli* has been reported to exhibit characteristic markers of apoptosis similar to those observed in multicellular organisms (Dwyer et al., 2012). Thus, we investigated the effect of anti-secretory agents on the NHPHs residing within the intracellular canaliculus of parietal cells, and the TUNEL method revealed apoptotic changes, following treatment with vonoprazan. We had speculated the apoptosis of the parietal cells having NHPH inside at first, but these changes could not be detected in our experiment.

Our present findings indicated a possible relationship between apoptotic NHPHs and TUNEL-positive bacteria. Further investigations are necessary to explore such a relationship.

## Data Availability

The raw data supporting the conclusions of this article will be made available by the authors, without undue reservation.
